# A Mechanistic Explanation Linking Adaptive Mutation, Niche Change, and Fitness Advantage for the Wrinkly Spreader

**DOI:** 10.1155/2014/675432

**Published:** 2014-01-16

**Authors:** Andrew J. Spiers

**Affiliations:** The SIMBIOS Centre & School of Science, Engineering and Technology, Abertay University, Bell Street, Dundee DD1 1HG, UK

## Abstract

Experimental evolution studies have investigated adaptive radiation in static liquid microcosms using the environmental bacterium *Pseudomonas fluorescens* SBW25. In evolving populations a novel adaptive mutant known as the Wrinkly Spreader arises within days having significant fitness advantage over the ancestral strain. A molecular investigation of the Wrinkly Spreader has provided a mechanistic explanation linking mutation with fitness improvement through the production of a cellulose-based biofilm at the air-liquid interface. Colonisation of this niche provides greater access to oxygen, allowing faster growth than that possible for non-biofilm—forming competitors located in the lower anoxic region of the microcosm. Cellulose is probably normally used for attachment to plant and soil aggregate surfaces and to provide protection in dehydrating conditions. However, the evolutionary innovation of the Wrinkly Spreader in static microcosms is the use of cellulose as the matrix of a robust biofilm, and is achieved through mutations that deregulate multiple diguanylate cyclases leading to the over-production of cyclic-*di*-GMP and the stimulation of cellulose expression. The mechanistic explanation of the Wrinkly Spreader success is an exemplar of the modern evolutionary synthesis, linking molecular biology with evolutionary ecology, and provides an insight into the phenomenal ability of bacteria to adapt to novel environments.

## 1. Introduction

Competition for limited resources and divergent selection arising from differences in the environment are key drivers of ecological adaptive radiation and ultimately speciation [1]. Although usually illustrated by reference to examples such as Darwin's finches in the Galapagos or the cichlid fishes in East African Rift Valley lakes [2–4], adaptive radiation has also played an important role in the great phylogenetic and functional diversification of bacteria and can help explain in part bacterial colonisation and niche preferences, as well as bacterial community complexity, interactions, and dynamics (bacterial adaptive radiation differs in some fundamental ways to that seen in sexual populations [4, 5]). Key to adaptive radiation is ecological opportunity which promotes adaptive radiation by changing the selective pressures acting on populations, relaxing stabilising selection and creating conditions that generate diversifying selection [6]. The rate at which bacterial populations become locally adapted depends on both the selective regime as well as the rate at which adaptive mutations arise and are fixed within the population. In bacteria, adaptations may arise through mutation of existing genomes and horizontal or lateral gene transfer (HGT) within populations or between phylogenetically similar or distant species. HGT is generally viewed as the primary means of acquisition of adaptive mutations for bacteria, whereas gene duplications are thought of as the main source of adaptive novelty in eukaryotes [7, 8]. Although bacterial adaptive radiation can be studied by phylogeographical analyses, it is also readily investigated by experimental evolution studies where genetic changes and fitness increases have been observed in relatively short periods (for reviews, see [9–14]).

### 1.1. Bacterial Evolution in Simple Microcosms

The use of simple microcosms for modelling bacterial evolution has been a successful approach because bacterial populations are readily grown *in vitro* where they can be initiated with small isogenic samples, have short generation times, and reach very large population sizes. During the development of these populations, mutations occur randomly and at sufficient rates, ensuring that large numbers of novel genotypes appear during the course of the study and are subjected to selective pressures, genetic drift, and stochastic events. These mutants are often easily identified by altered phenotypes determined by growth on agar plates or by simple assays, and individual mutants and population samples can be indefinitely stored at −80°C, allowing comparisons to be made across time points and between ancestral and evolved strains. In particular, this allows testing of fitness changes, where adaptive genotypes are defined as having a competitive fitness (*W*) advantage over the ancestor. *W* can be calculated as the ratio of Malthusian parameters for continuously growing populations: when *W* > 1, the mutant has a fitness advantage over the ancestor and is considered an adaptive genotype; when *W* = 1, the two strains are neutral with respect to one another; and when *W* < 1 the mutant is at a selective disadvantage [15–17].

If the possibility of HGT is excluded in experimental populations, the origin of all adaptive mutations must be through alterations to the ancestral bacterial genome (chromosome and accompanying plasmids) and might range from small-scale sequence changes affecting a single gene to larger rearrangements including deletions or duplications of whole operons or considerable portions of the genome. These can be identified through sequencing target genes or through whole genome resequencing, and the underlying molecular biology of individual genotypes can be further investigated in terms of gene expression patterns, regulatory networks, and metabolism, in order to understand how adaptive mutations can be mechanistically linked to altered phenotypes and fitness changes [18].

Although experimental microcosms tend to be physically and chemically simple, they can be manipulated to change ecological opportunities, competition, environmental conditions, and selective pressures. For example, glass vials containing liquid growth medium can be incubated with constant shaking to provide a homogeneous environment, or statically where spatial structure becomes important with the microcosm becoming heterogeneous and containing new niches at the air-liquid interface, the liquid column, and vial bottom [19]. Nutrient type, abundance, and complexity, as well as the chemical environment (e.g., osmolarity, O_2_, and pH), can all be manipulated through changes to the growth medium. The small size, low cost, and reproducibility of such microcosms mean that they can be used in large numbers with appropriate levels of replication, allowing multifactorial experimental design and including the ability to repeat experiments using exactly the same initial conditions when required.

### 1.2. Some Molecular Aspects Underlying Bacterial Evolution

In comparison with highly specialized bacteria such as pathogens and symbionts, generalists have a high proportion of sensory and regulatory systems which allow them to respond to a wide range of environmental factors and opportunities. These presumably arose through distant gene duplication and acquisition events and often involve multifunctional proteins linking sensory and signal transduction domains which retain little homology beyond the functional domains themselves. While some regulatory elements act to modify cellular response or homeostasis *via* transcription, others modify activity through secondary signal molecules such as cyclic-*di*-GMP (bis-(3′-5′)-cyclic dimeric guanosine monophosphate). Cyclic-*di*-GMP is an intracellular signalling molecule that plays a central role in the regulation of motility, virulence, and biofilm formation in many bacteria (for reviews, see [20–24]). It is synthesised from GTP (guanosine-5′-triphosphate) by DGCs (di-guanylate cyclases) and hydrolysed by specific PDEs (phosphodiesterases) to guanosine monophosphate (GMP) or 5′-pGpG (linearized *di*-GMP) which is subsequently hydrolysed to GMP by other hydrolases. DGCs are characterised by the amino acid motif Gly-Gly-Asp-Glu-Phe, referred to as the GGDEF domain, whilst PDEs include the Glu-Ala-Leu (EAL) or His-Asp-x-Gly-Tyr-Pro (HD-GYP) domains.

Bacterial genomes tend to have multiple DGCs and PDEs which suggests that cyclic-*di*-GMP levels are regulated through a complex signaling network integrating numerous environmental signals that control riboswitches, transcription factors, and enzyme activities including cellulose expression. The disruption of homeostasis through mutation of a high-level sensory-regulator systems can have a significant impact on bacterial phenotype, allowing significant fitness leaps to occur rather than the expected smaller incremental steps. Furthermore, the duplication and divergence of regulators can lead to significant reprogramming of regulatory networks [25], with transferred genes (xenologues) generally persisting in genomes longer than duplicated genes (paralogues) which tend to have more protein-protein interactions and regulators [8].

## 2. Adaptive Radiation of *P. fluorescens* in Static Microcosms

The soil and plant-associated fluorescent pseudomonad, *Pseudomonas fluorescens* SBW25, has been used as a model bacterium in experimental evolution studies using simple microcosms [19]. SBW25 was originally isolated from the leaf of a sugar beet (*Beta vulgaris*) plant [26] and is capable of colonising a wide variety of crop plants and weeds. Like other fluorescent pseudomonads, it is regarded as a benign, plant growth-promoting rhizobacterium (i.e., one that grows on or around the roots of plants in the rhizosphere). However, SBW25 carries a defective pathogen-like type III secretion system [27] and expresses the virulence-associated cyclic lipopeptide class surfactant, viscosin [28]. Many *P. fluorescens* strains including SBW25 produce soft rot-like symptoms when colonising plant tissues following physical damage, suggesting that these soil and plant-associated bacteria are highly competent colonists with opportunistic pathogenic tendencies. The ability to colonise new environments is a characteristic associated with the pseudomonads and may be enabled by relatively large genomes that include many sensory and regulatory elements [29–31]. SBW25 sequences were initially maintained in a partial genomic database before the whole genome sequence was determined [30, 32], and comprehensive molecular analyses of regulatory pathways, metabolism, and fitness are possible using techniques established for other pseudomonads. In addition to experimental evolution studies, SBW25 has been used to study plant-microbe interactions (e.g., [30, 33, 34]), air-liquid (A-L) interface biofilms, and cellulose expression (e.g., [35–39]).

**Figure 1:**
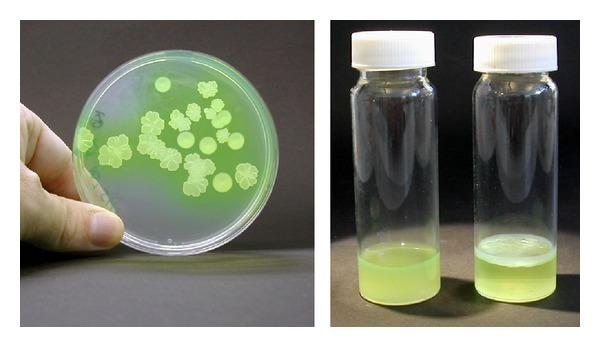
*Adaptive radiation in static microcosms gives rise to the Wrinkly Spreader with a new niche preference*. Shown on the left is a King's B agar plate, incubated for three days at 28°C, spread with a sample taken from a diversified *P. fluorescens* SBW25 population where both Smooth morphs and Wrinkly Spreader colonies are evident (this plate has seven Smooth colonies each with a rounded circumference and a smooth convex surface, with one positioned at the top of the plate; all of the rest are Wrinkly Spreader colonies which have irregular, multilobed circumferences and a flattened and wrinkled surface). On the right are two static King's B microcosms which were incubated for three days at 28°C. The left microcosm was inoculated with the wild-type or ancestral SBW25 which grows throughout the liquid column, and the right microcosm with the Wrinkly Spreader which colonises the A-L interface through the formation of a robust biofilm.

In experimental evolution studies, SBW25 populations have been maintained in small glass vials containing King's B liquid growth medium [40] which is incubated statically or with shaking (these are 30 ml universal vials containing 6ml medium; see Figure 1). The adaptive radiation of SBW25 in static microcosms is highly reproducible with populations diversifying over 3–5 days to produce a range of phenotypically distinguishable genotypes occupying different niches [19] (it is notable that in significantly smaller microcosms, SBW25 diversification is far less reproducible, suggesting that the reproducibility is due in part to larger population sizes and numbers of mutants produced in the larger microcosms [19]). The main genotypes are the Smooth morphs (an abbreviation of morphotypes) which produce round, smooth colonies on King's B agar plates and colonise the liquid column of King's B medium within static microcosms (these include the ancestral or wild-type SBW25), the Fuzzy Spreaders which produce stippled colonies and appear to colonise the bottom of static microcosms, and the Wrinkly Spreaders which produce a wrinkled colony morphology and colonise the A-L interface or surface of the liquid column through the formation of a visually obvious and robust biofilm (see Figure 1) (the Wrinkly Spreader phenotype is also observed using other growth media such Luria Bertani and minimal glucose media). These main genotypes are also referred to as ecomorphs to reflect their niche specialisations within the static microcosm. As SBW25 reproduction is entirely asexual in static microcosms (SBW25 does not carry a self-transmissible plasmid nor any other mobile genetic element), these genotypes are analogous to species [41]. Variation also occurs within genotypes, and each can further diversify to produce the other genotypes, though at reduced levels compared to ancestral SBW25 [42].

A simplistic explanation of the diversification and adaptation of SBW25 in static microcosms is provided by the Red Queen hypothesis which demands constant evolution in response to ever-changing competitors and environments [43, 44]. Competitive interactions between the Smooth, Fuzzy Spreader and Wrinkly Spreaders result in the stable maintenance of diversity. In most cases, populations dominated by one genotype can be invaded by a rare genotype which can colonise an unoccupied niche, and this type of competitive tradeoff between niche specialists is frequency dependent. For example, the Wrinkly Spreader can invade a population of ancestral SBW25 by colonising the A-L interface (*W* = 1.46), whilst the ancestral SBW25 can invade a population of Wrinkly Spreaders by colonising the liquid column (*W* = 1.66) (in five of the six pairwise combinations, the rare genotype can invade the common one; however, the Fuzzy Spreader is unable to invade the Wrinkly Spreader) [19]. Furthermore, fitness differences between independently isolated Wrinkly Spreaders suggest that there can be strong competition within the biofilm itself, and as the biofilm gets older, later arising Wrinkly Spreaders have greater fitness than earlier isolates even though the diversity within the genotype decreases [45–48].

## 3. Rise of the Wrinkly Spreaders

In wild-type SBW25 populations developing in King's B static microcosms, Wrinkly Spreaders will appear through random mutation and can represent up to 30–50% of the total population after five days when biofilms are usually evident [19, 49, 50]. The mutation rate of ~10^−7^ mutations/cell/generation is not unusually elevated in static microcosms, and Wrinkly Spreaders also appear in shaken microcosms where they might represent ~10% of the population after the same period.

When individual Wrinkly Spreader isolates, recovered by spreading microcosm samples on King's B agar plates and selecting single colonies, are reintroduced into King's B static microcosms, they produce detectable biofilms covering the entire A-L interface within twelve hours that continue to develop over 3–5 days, often reaching 1–1.5 mm in depth and containing ~10^6^ cells/mL, before breaking and sinking [51]. A-L interface biofilms are sometimes referred to as pellicles in contrast to the archetypal L-S (liquid-solid surface) interface biofilms investigated using flow cells and confocal laser scanning microscopy [52], though biofilms at the meniscus and A-L interface of static liquids form a continuum of structures that link A-L, A-L-S (air-liquid-solid surface) and L-S biofilms which can be quantitatively differentiated [13, 39]. Unlike static microcosms containing mixed genotype SBW25 populations in which biofilm material at the A-L interface and substantial growth in the liquid column is evident, Wrinkly Spreader static microcosms show very little growth in the liquid column below the biofilm which often appears clear.

The rise of the Wrinkly Spreader in static microcosms is explained by the fitness advantage (*W* = 1.5–2.5) these genotypes have over non-biofilm-forming competitors including the ancestral SBW25 (the range of *W* values reflects differences in the assay conditions and the choice of reference strain) [19, 35, 49]. The evolutionary innovation of the Wrinkly Spreader is the production of a biofilm located at the A-L interface of static microcosms, sufficient to withstand the normal spectrum of physical disturbances (i.e., vibrations and random knocks), which allows better access to O_2_ diffusing from the atmosphere into the liquid column. As a result, the Wrinkly Spreader shows a new niche preference compared to the ancestral SBW25.

The SBW25 colonists establish an O_2_ gradient within three hours, defining an O_2_-rich upper zone of ~200 *μ*m and a lower O_2_-depleted anoxic zone of ~16 mm in King's B static microcosms which persist for up to five days [50]. The O_2_-rich conditions of the upper zone support higher rates of growth, as SBW25 growth is O_2_ rather than nutrient-limited in King's B medium, increasing the chance that a Wrinkly Spreader mutant will arise in the developing population. Wrinkly Spreader cells that are recruited to the A-L interface will grow faster than non-biofilm-forming competitors that cannot maintain a presence in the O_2_-rich zone and quickly form a biofilm and further repress the growth of competitors. Growth conditions in the biofilm have a significant impact on the physiology of SBW25 cells, as biofilm-isolated cells can be differentiated from those recovered immediately below the biofilm by Raman spectral profiling [53].

**Figure 2:**
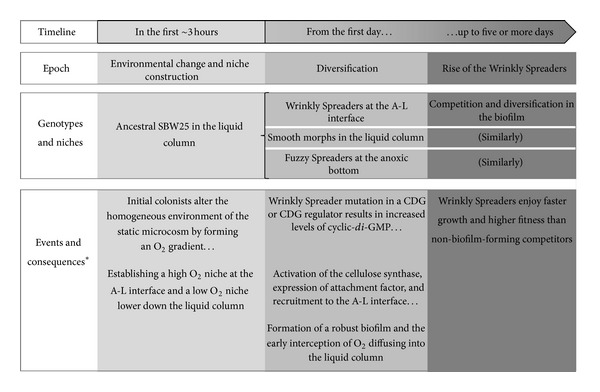
*Linking the timeline of adaptive radiation to events and consequences in the rise of the Wrinkly Spreader*. The adaptive radiation of *P. fluorescens* SBW25 in static microcosms can be mapped to a timeline and epochs during which the ancestral strain diversifies into new genotypes with altered niche preferences. The events and consequences of mutation are outlined for the Wrinkly Spreader along the bottom panel of the figure. Although not shown here, further diversification will occur amongst the Smooth morphs and Fuzzy Spreaders.

Although both the ancestral SBW25 and the Wrinkly Spreader can swim using flagella, the successful recruitment of the Wrinkly Spreader probably is the result of altered cell surface charge or relative hydrophobicity [54], and once located at the meniscus region close to the vial walls, Wrinkly Spreader cells also show a higher level of attachment than the ancestral SBW25 [51, 54]. In this system, SBW25 populations are altering their environment through niche construction, and these changes feedback to influence the subsequent evolution of the Wrinkly Spreaders through ecoevolutionary (ecological-evolutionary) feedbacks where the time scales of environmental change and evolution are similar [55, 56] (see the timeline of events in the diversification of SBW25 populations in static microcosms leading to the rise of the Wrinkly Spreaders in Figure 2).

The Wrinkly Spreader biofilm probably develops from microcolonies attached at the meniscus which grow out across the A-L interface, and once the liquid surface is covered, the biofilm develops further by continued growth at the top surface, slowly displacing the lower region of the biofilm further into the liquid column [50, 57]. This development requires the cooperation of the growing Wrinkly Spreader population, and the biofilm is the result of the clonal expansion of a mutant lineage expressing the primary biofilm matrix material, cellulose, rather than the result of quorum-based regulation of extracellular polymeric substance or exopolysaccharide (EPS) expression often required for other biofilms [52]. This cooperation is explained by Hamilton's inclusive fitness or kin selection theory, which states that cooperation evolves between genetically related individuals [58], and the view of the biofilm structure as a common good that is shared by all members of the community is supported by the finding that non-biofilm-forming cheaters also appear in Wrinkly Spreader biofilms [59].

Ultimately, the fitness advantage of the Wrinkly Spreader is attributable to better O_2_ access through the formation of a biofilm at the A-L interface [50]. Although the development of the biofilm is the result of the cooperation of many generations of Wrinkly Spreaders, it can also be viewed as a selfish trait (of the Wrinkly Spreader lineage) and possibly an example of ancestor's inhibition, as the constant production of EPS pushes later generations of cells upwards towards better O_2_ conditions and older generations downwards into the anoxic region where growth is limited [60, 61]. In contrast, in shaken microcosms where no O_2_ gradients can be established, the Wrinkly Spreader has a lower fitness compared to non-biofilm-forming competitors or the ancestral SBW25 (*W* = ~0.3–1.0) [35, 49]. Furthermore, on King's B agar plates where the Wrinkly Spreader is genetically unstable and rapidly generates Smooth-like revertants (phenotypically similar to the biofilm cheaters recovered from static microcosms), the Wrinkly Spreader has an even lower fitness (*W* = 0.15) [62].

## 4. A Mechanistic Explanation for the Wrinkly Spreader

The molecular biology underlying the Wrinkly Spreader (WS) phenotype was first investigated using a mini-Tn*5* transposon screening approach in order to identify critical genes and regulatory pathways required for a wrinkled colony and A-L interface biofilm formation [35] (the archetypal Wrinkly Spreader referred to here is a specific strain also recorded as PR1200 [35] and Large Spreading Wrinkly Spreader (LSWS) [46]). Two sets of Wrinkly Spreader mini-Tn*5* mutants were recovered and used to characterise the *wsp* and *wss* operons responsible for the regulation and production of the WS phenotype, respectively [35, 46, 51, 54, 63, 64].

### 4.1. From the *wsp* Regulatory Operon to the Mutations that Activate the WS Phenotype

**Figure 3:**
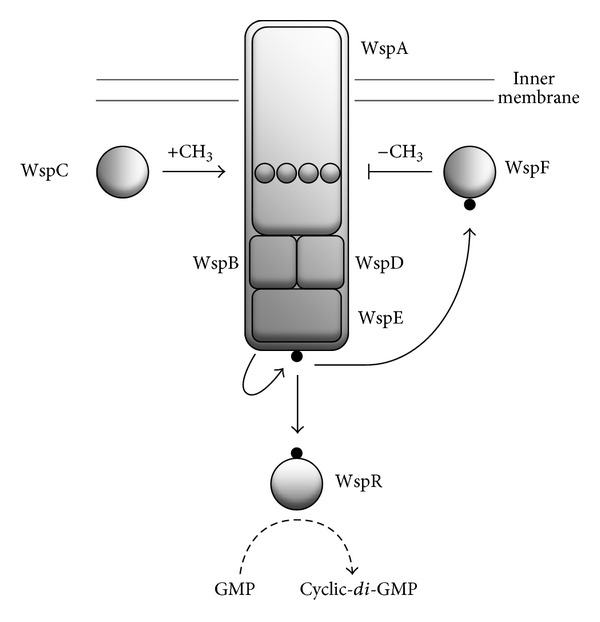
*The Wsp system is responsible for the synthesis of cyclic-di-GMP and the activation of the Wrinkly Spreader (WS) phenotype*. The functioning of the *P. fluorescens* SBW25 Wsp system has been modelled on the Che chemosensory system of *E. coli* and provides a mechanistic explanation linking adaptive mutations to the WS phenotype. The methyl-accepting chemotaxis protein (WspA), scaffold proteins (WspB and WspD), and histidine kinase (WspE) form a membrane-associated receptor-signaling complex. In the absence of an appropriate environmental signal, the complex is silent. Upon activation by phosphorylation (indicated by the black circles), the diguanylate cyclase (DGC) response regulator (WspR) synthesizes cyclic-*di*-GMP from GTP. The system is controlled by the opposing activities of a methyltransferase (WspC) and methylesterase (WspF), which add and remove, respectively, methyl (CH_3_) groups on the signalling domain of WspA (circles). In wild-type SBW25, the activities of the two are balanced, preventing the activation of WspR and allowing the Wsp complex to oscillate between active and inactive states. Mutations inhibiting WspF function or activating WspE kinase activity result in the activation of WspR and the production of cyclic-*di*-GMP. Increased levels of cyclic-*di*-GMP then lead to the expression of the WS phenotype. The Wsp system is shown as a schematic only; the three-dimensional structure of the proteins, their relative placement, numbers, and the positioning of the complex in the inner membrane have not yet been determined.

The *wsp* regulatory operon consists of seven genes (*wspA-F and wspR*) showing significant protein level homology with chemosensory signal-transduction-like operons (*wsp* is an acronym for WS phenotype, reflecting the fact that this operon encodes a regulatory component required for the WS phenotype) [35, 46]. The functioning of the Wsp system (see Figure 3) has been modelled on the Che chemosensory system of *Escherichia coli* [65] to provide a mechanistic explanation of the induction of the WS phenotype [46]. Based on the behaviour of the homologous Wsp proteins in *P. aeruginosa* PA01 [66], WspA, WspB, WspC, WspD, WspE, and WspF are likely to form a membrane-associated receptor-signaling complex that responds to stimulus associated with the growth on a solid surface [67] by activating the associated WspR response regulator (SBW25 *wsp*-like operons are also found in the related pseudomonads, *P. fluorescens* Pf0-1, *P. putida* KT2440, and *P. syringae* pv *tomato *DC3000; see the *Pseudomonas *Genome Database [68]).

WspR is a DGC which catalyses the synthesis of cyclic-*di*-GMP when phosphorylated [64]. In wild-type SBW25, WspR activity is modulated by the opposing activities of the WspC and WspF subunits. However, *wspF* mutations that are predicted to reduce or abolish WspF function have been identified in a number of independently isolated Wrinkly Spreaders [46]. Allele-exchange experiments have shown that a *wspF* mutation is necessary and sufficient for the WS phenotype, converting wild-type SBW25 into a Wrinkly Spreader. Conversely, the replacement of a *wspF* mutation with the wild-type sequence reverts the strain back to the ancestral phenotype [46]. Subsequently, *wspE* mutations have also been identified in independently isolated Wrinkly Spreaders [69]. In these, the activity of WspE may be increased leading directly to the overactivation of WspR. Perhaps surprisingly, no *wspR* mutations have been identified in independently isolated Wrinkly Spreaders to date. However, mutations affecting two other DGCs, AwsR and MwsR, and a putative DGC-PDE hybrid, SwsR, are also known to induce the WS phenotype [34, 69–71]. Although three different DGCs appear to be targeted by adaptive mutation, it is noteworthy that the SBW25 genome contains thirty-nine putative genes encoding DGCs [30]. Similarly, in Pf0-1, four of thirty putative DGCs appear to be involved in biofilm formation [72]. Systematic analyses of these show that one DGC preferentially affects the localisation of the primary Pf0-1 protein adhesin, LapA, another controls swimming, and the last affects both LapA and motility. These findings suggest that different cyclic-*di*-GMP-regulated systems can be specifically controlled by distinct DGCs [72].

### 4.2. The Involvement of Cellulose in the WS Phenotype

The cyclic-*di*-GMP catalysed by WspR or other DGCs in a number of independently isolated Wrinkly Spreaders is believed to activate a membrane-associated cellulose synthase complex encoded by the ten gene *wss* operon (*wssA-J*) (*wss* is an acronym for WS structural, reflecting the fact that this operon encodes a structural component required for the WS phenotype) [35]. WssB, WssC, WssD, and WssE show significant protein level homology to the core cellulose synthase subunits originally identified in the *bcs* (bacterial cellulose synthesizing) operons of *Acetobacter xylinum *(now known as *Gluconacetobacter hansenii *ATCC 23769, [73]) and *E. coli* K-12 [74], with WssB identified as the cyclic-*di*-GMP-binding catalytically active subunit responsible for the polymerisation of UDP glucose into cellulose [35]. However, the *wss* operon contains additional genes (*wssA* and wss*F-J*) not previously recognised as having a role in cellulose synthesis. WssA and WssJ are MinD-like homologues and may be responsible for the correct spatial localization of the Wss cellulose synthase complex at the cell poles as is the case for the K12 YhjQ-BcsQ WssA homologue [75], and WssF is predicted to provide acyl groups to WssG, WssH, and WssI which share homology with the AlgF, AlgI, and AlgJ alginate acetylation proteins of *P. aeruginosa* FRD1 [76].

Although *bcs* operons are widespread amongst bacteria [39], only DC3000 has a complete *wss* operon including the acetylation-associated genes *wssF-I*, whilst KT2440 has a truncated *wssA-E* operon (see the *Pseudomonas* Genome Database [68]), and both have been shown to express cellulose experimentally [36] (in contrast, PA01 and Pf0-1 do not contain *bcs *operons and utilise other matrix components in their biofilms [77, 78]).

The expression of extracellular cellulose by the Wrinkly Spreader was confirmed by comparative Congo red staining of colonies, Calcofluor-based fluorescent microscopy, and cellulase digestion of biofilm material and was chemically identified as partially acetylated cellulose by the structural analysis of purified biofilm matrix material [35, 51] (reviewed by [39]). Significantly, a Wrinkly Spreader *wspR* mini-Tn*5* mutant does not express cellulose, whilst the addition of a plasmid-borne constitutively active WspR mutant in wild-type SBW25 expresses cellulose and produces the WS phenotype [35, 51, 54, 63]. Further minitransposon analysis of the Wrinkly Spreader has confirmed that all of the *wsp* and *wss* genes, except *wssJ*, are required for the WS phenotype and that WssJ may be functionally redundant [70].

The Wrinkly Spreader biofilm matrix appears as an extensive network of extracellular cellulose, with 0.02–100 *μ*m thick fibres forming thin films around voids and linking large clumps of material [51]. The structure itself is highly hydrated, containing 97% liquid and having a density almost equivalent to that of the culture media. Within the biofilm, bacterial cells are associated with the cellulose fibres and are also found within the voids [51]. Scanning electron microscopy and confocal laser scanning microscopy suggest that the biofilm may be a lattice work of pores produced by constant growth at the top surface of the biofilm which slowly displaces older strata deeper into the liquid column [39, 50]. The degree of wrinkleality (or wrinkledness) [13] of Wrinkly Spreader biofilms and colonies depends on interactions between the cellulose fibres and cells, as well as lipopolysaccharide and an unidentified fimbriae-like attachment factor also overexpressed by the Wrinkly Spreader [54]. Wrinkleality can be quantified using a combination of assays, including colony expansion, reversion rates, growth in static microcosms, biofilm attachment levels, and strength [38, 51, 62] and used to differentiate between Wrinkly Spreader isolates (unpublished observations, A. Spiers & Y. Udall).

### 4.3. The Wrinkly Spreader Is Not the Only Genotype Capable of Exploiting the A-L Interface

In addition to the Wrinkly Spreader, SBW25 has been observed to produce two additional A-L interface biofilms. When induced nonspecifically with iron (FeCl_3_), wild-type SBW25 will produce a fragile, cellulose-based viscous mass (VM) biofilm which is poorly attached at the meniscus and substantially weaker than the Wrinkly Spreader biofilm [37]. This physiologically induced biofilm is phenotypically indistinguishable from that produced by JB01, a SBW25 strain in which the neomycin phosphotransferase gene promoter (*nptII*) was inserted upstream of the *wss* operon to increase transcription and cellulose expression [35] (wild-type SBW25 expresses low levels of cellulose when grown in King's B medium, suggesting that there is some cyclic-*di*-GMP available to induce the cellulose synthase complex [35, 51]). This suggests that mutants that produce VM-like biofilms are likely to appear in diversifying populations of wild-type SBW25, perhaps increasing *wss* promoter activity directly or indirectly by reducing the functioning of *wss* transcriptional repressors. It is possible that such mutants have escaped attention, as they would be expected to produce Smooth-like colonies similar to those produced by JB01.

Complementary biofilm-forming strain (CBFS) mutants of a cellulose-deficient SBW25 strain have also been isolated from static microcosms, though for these, the mechanism underlying biofilm-formation is as yet unknown [70]. A comparison of all three biofilm types using a common non-biofilm-forming reference strain indicates that all provide a fitness advantage in static microcosms (*W* = 2.7, 2.2, and 1.8 for CBFS, VM, and WS, resp.) and can be differentiated on the basis of a number of quantitative biofilm-associated assays, suggesting that they represent three different solutions to the colonisation of the A-L interface (unpublished observations, A. Spiers & A. Koza). It is not yet clear why the Wrinkly Spreader appears to be the most successful biofilm type arising in diversifying populations of SBW25. However, it is possible that the biofilms produced by CBFS or VM-like mutants may be more costly, physically unreliable, or structurally overengineered compared to the Wrinkly Spreader. Alternatively, the genetic architecture of SBW25 may favour mutations that result in the WS phenotype rather than a partial VM phenotype where only cellulose expression is activated, or the activation of an entirely different pathway leading to the CBFS phenotype.

## 5. Possible Role of Cellulose in Natural Environments

Although the ability to produce cellulose-based A-L interface biofilms in static microcosms is common amongst environmental pseudomonads [36, 38, 79] (reviewed by [39]), it is unclear what the functional role of cellulose might be in the natural habitats of these bacteria. Whilst biofilm-formation is a key bacterial strategy for colonisation, it is only one of a range of assemblages that bacteria can form, ranging from isolated surface-attached bacteria, microcolonies, and multilayered, multispecies, and differentiated biofilms to flocs and slime (for reviews, see [52, 78, 80–84]). In contrast to the view of biofilm formation as a genetically determined developmental programme [85], biofilms are transient communities better described by adaptation, social evolution, and ecological succession [58, 81, 86]. In these, the Red Queen may drive competition and adaptation in nascent biofilms, but the Black Queen may play a greater role in developing longer lasting and more robust interdependent cooperative communities in older, more permanent structures [87, 88].

It is possible that biofilm-formation is used by environmental pseudomonads to rapidly colonise the meniscus and A-L interface of temporary water bodies such as those found in partially saturated soil pore networks or collected on surfaces after rainfall. However, the paradigm of the immersed cellulose matrix-based biofilm exemplified by the Wrinkly Spreader is challenged by the finding that cellulose expression by SBW25 provides a fitness advantage in natural environments where water does not collect or remain for any length of time. Competitive fitness assays have shown that wild-type SBW25 has a fitness advantage compared to a cellulose-deficient mutant of *W* = 1.8 on the leaves of sugar beet seedlings and *W* = 1.1 on the roots [33]. Recent experimentation has shown that SBW25 colonizes mushroom (*Agaricus bisporus*) caps where it can produce blotch-like disease symptoms similar to other *Pseudomonas* spp. mushroom pathogens, and in these situations the ability to express cellulose also provides a fitness advantage of *W* = 1.2–1.4 (unpublished observations, A. Spiers & A. Koza). An alternative role for cellulose in these natural, nonsaturated environments is suggested by the finding that cellulose expression also enhances the survival of SBW25 under dehydrating or low humidity conditions (unpublished observations, A. Spiers & A. Koza), where cellulose fibres might help to retain water around microcolonies and trap water vapour directly from the air [82, 89]. Conceivably the cellulose matrix may also help with nutrient acquisition (e.g., from root exudates and soil aggregates) and retention.

## 6. Concluding Statement

Even in simple microcosms, ecological opportunity and competition between genotypes can act to drive the adaptive radiation of bacterial populations. In the case of SBW25, it appears that the presence of multiple DGCs in the genome, resulting from gene duplications and acquisitions deep in the phylogenetic history of the strain, predisposes it to adaptive mutation, niche change, and fitness leaps in static liquid microcosms, leading to the rise of the Wrinkly Spreaders. The molecular biology underlying the WS phenotype is now well understood, providing a mechanistic explanation linking adaptive mutations which activate DGCs to overproduce cyclic-*di*-GMP, the expression of cellulose, and the formation of biofilms at the A-L interface, with the fitness benefit obtained by the colonisation of this new niche over non-biofilm-forming competitors and the ancestral SBW25 strain.
